# Ultrafast Chiral Precession of Spin and Orbital Angular Momentum Induced by Circularly Polarized Laser Pulse in Elementary Ferromagnets

**DOI:** 10.1021/acs.jpclett.4c00291

**Published:** 2024-02-26

**Authors:** Junjie He, Thomas Frauenheim, Shuo Li

**Affiliations:** † Department of Physical and Macromolecular Chemistry, Faculty of Science, Charles University, Prague 12843, Czech Republic; ‡ Bremen Center for Computational Materials Science, University of Bremen, Bremen 28359, Germany; § Institute for Advanced Study, Chengdu University, Chengdu 610106, China

## Abstract

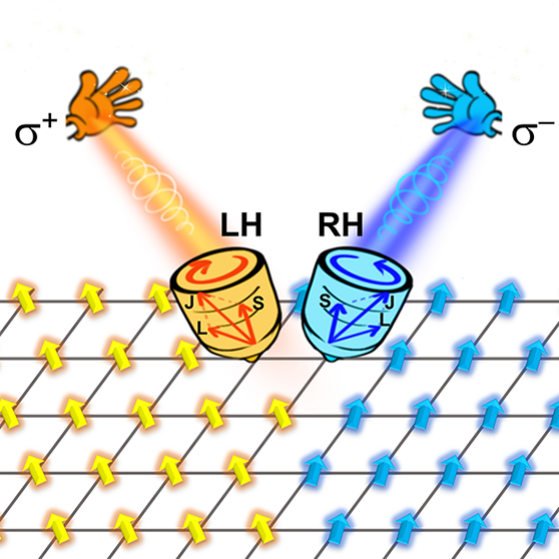

Despite spin (SAM) and orbital (OAM) angular momentum dynamics being well-studied in demagnetization processes, their components receive less focus. Here, we utilize real-time time-dependent density functional theory (rt-TDDFT) to unveil significant *x* and *y* components of SAM and OAM induced by circularly left (σ^+^) and right (σ^–^) polarized laser pulses in ferromagnetic Fe, Co, and Ni. Our results show that the magnitude of the OAM is an order of magnitude larger than that of the SAM, highlighting a stronger optical response from the orbital degrees of freedom of electrons. Intriguingly, σ^+^ and σ^–^ pulses induce chirality in the precession of SAM and OAM, respectively, with clear associations with laser frequency and duration. Finally, we demonstrate the time scale of the OAM and SAM precession occurs even earlier than that of the demagnetization process and the OISTR effect. Our results provide detailed insight into the dynamics of SAM and OAM during and shortly after a polarized laser pulse.

Beaurepaire et al.^1^ made a groundbreaking discovery, showcasing ultrafast demagnetization of ferromagnetic nickel within a subpicosecond time scale through femtosecond laser pulses—a process 3 orders of magnitude faster than that achievable with magnetic fields alone. This discovery not only has profound implications for fundamental science but also heralds potential breakthroughs in technological applications.^2,3^ With advancements in generating ultrashort laser pulses, the time scale for spin manipulation has now reached femtosecond and even attosecond domains.^4−7^ Recently, Dewhurst et al.^8^ proposed a new mechanism for ultrafast spin manipulation, termed the optically induced intersite spin transfer (OISTR) effect, which has since received experimental corroboration.^9−13^ The OISTR effect demonstrates that optical excitation can coherently and efficiently redistribute spins among distinct magnetic sublattices within tens to a mere few femtoseconds, positioning it as the most rapid method for controlling spin in magnetic materials.^7^

While the OISTR effect originates from light-induced spin-dependent charge excitation on femtosecond time scales and predominantly emphasizes the magnitude of spin transfer and its consequent demagnetization,^7,8^ it does not extensively address the changes in spin component. This leads to pertinent questions: (i) How does the laser pulse influence the spin angular momentum (SAM) components during the OISTR effect? (ii) What role does the orbital angular momentum (OAM) play in the OISTR process? Addressing these questions is of paramount importance to gain a comprehensive understanding of the intricacies involved in the OISTR effect and its potential implications for ultrafast magnetization phenomena.

Spin–orbit interactions play a fundamental role in the dynamics of demagnetization processes. This relativistic interaction breaks the conservation of the total electronic SAM (defined as **S**), potentially leading to a transfer between SAM and OAM (defined as **L**).^14,15^ Such transfers are critical processes that determine the speed of the ultrafast magnetization phenomena. However, despite its paramount importance, the experimental evidence on the interchange between SAM and OAM remains ambiguous, as highlighted by studies using magnetic circular dichroism (MCD) and X-ray absorption spectra (XAS).^16−20^ For instance, Boeglin et al.^21^ indicate that OAM might change even faster than spin in experiments. Conversely, recent theoretical studies offer insights supporting the transfer of SAM into OAM.^22^

Much of the research has primarily concentrated on quantifying the transfer of SAM and OAM—demonstrating an initial angular momentum flow of **L** and **S**, which holds potential for experimental observation.^19−21^ However, the temporal evolution of the components of SAM and OAM, especially the *x* and *y* components, has been overlooked for a long time. The components of SAM could be brought about by photoinduced spin-polarized currents, and the OAM of electrons is gaining prominence in the realm of orbitronic devices.^23−25^ Pivoting back to the core issue of demagnetization dynamics, several important questions remain unanswered: (i) How do the directions of the SAM and the OAM evolve during the demagnetization process? (ii) What is the time scale for components of SAM and OAM? (iii) How do their respective components of angular momentum interact and transfer via spin–orbit coupling (SOC)? Addressing these questions is crucial for a comprehensive understanding of the microscopic processes underlying demagnetization dynamics.

To address the aforementioned issues, in this work, we present a detailed study of the time-dependent components of SAM and OAM dynamics under the influence of a circularly polarized laser pulse utilizing first-principles calculations. We proposed the concept of the chirality of spin and orbital precession and subsequently dissected the disparity in precession angles between the OAM and SAM. Furthermore, we explored the influence of laser parameters on the precession for the OAM and SAM.

We employed by a fully noncollinear spin version^26^ of rt-TDDFT by implementing through the full-potential augmented plane-wave ELK code (see https://elk.sourceforge.io/) to study the spin and orbital dynamics of Fe, Co, and Ni metals. Previous applications of rt-TDDFT have successfully elucidated ultrafast spin dynamics in metals, Heusler compounds, metallic alloying, and 2D magnets.^7−10,27−30^ The laser pulses employed in our analyses were circularly left (σ^+^) and right (σ^–^) polarized (see the computational details in the Supporting Information). We first consider a multilayer system of ferromagnetic Fe, Co, and Ni, consisting of a total of six monolayers (ML). Subsequently, a circularly polarized σ^+^ and σ^–^ pulse with a duration (FWHM) of 9.68 fs, a 1.63 eV (395 THz) frequency, and a fluence of 7.1 mJ/cm^2^ was employed to excite the multilayer system. While the time scale for this laser pulse aligns with that employed in the OISTR experiment as referenced in ref (7), it notably remains considerably shorter than the durations typically utilized in experiments probing the distinct responses of SAM and OAM dynamics. The ground-state SAM and OAM of the top layer of Co are presented in Figure 1, accompanied by the temporal evolution of these moments under the influence of the σ^+^ and σ^–^ pulses. We exclusively showcase the SAM and OAM dynamics in the top ML due to the similar properties exhibited by the other ML. The other ML exhibits similar properties.

**Figure 1:**
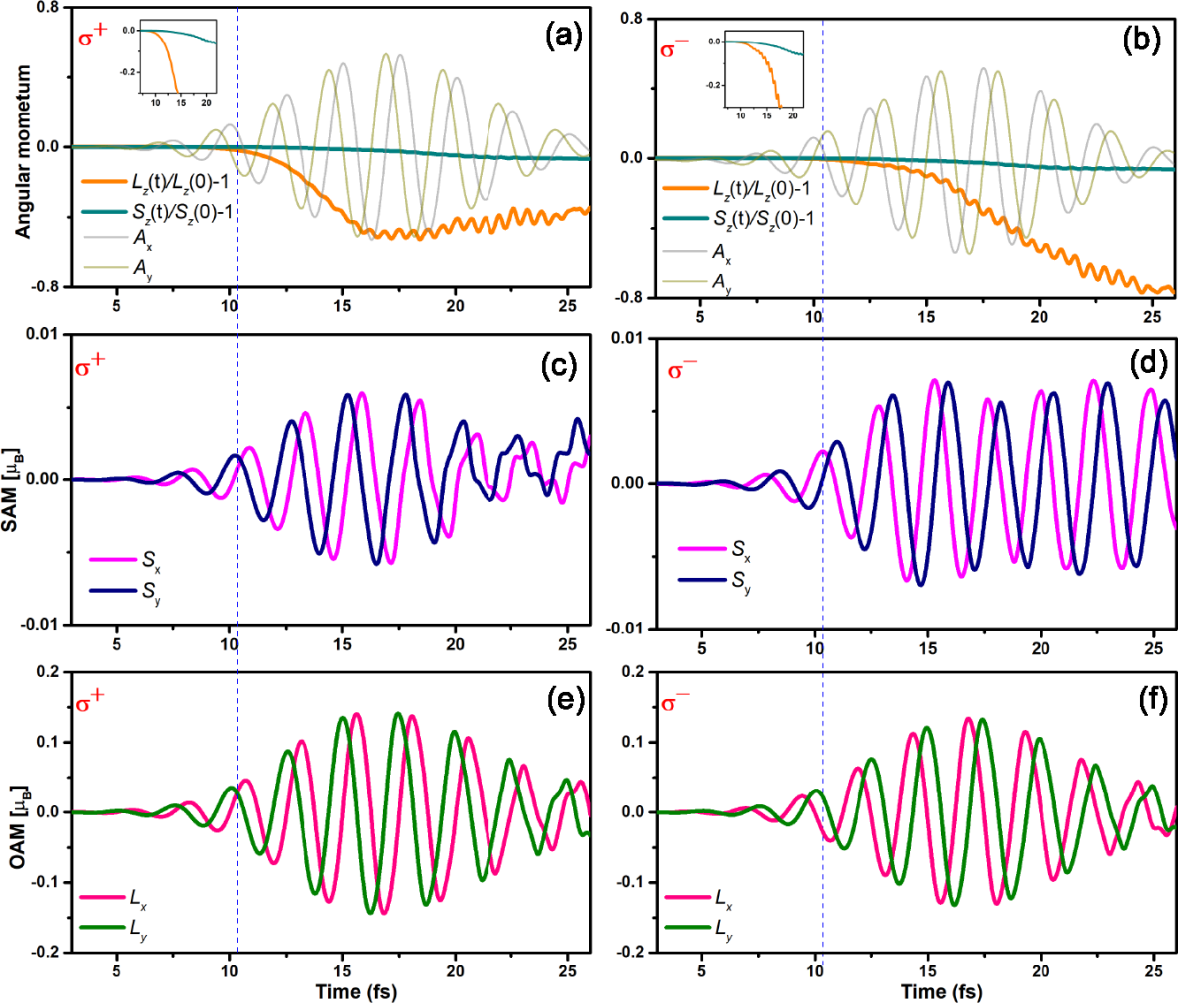
SAM and OAM dynamics are presented for the *x*, *y*, and *z* components under pulse excitations. The pulses are polarized with a full width at half-maximum (FWHM) of 9.68 fs, a central frequency of 1.63 eV, and an incident fluence of 3.55 mJ/cm^2^. (a) Normalized orbital angular momentum, *L*_*z*_(*t*)/*L*_*z*_(0) – 1, and spin angular momentum, *S*_*z*_(*t*)/*S*_*z*_(0) – 1, are depicted for Co. The light gray and yellow oscillating lines correspond to the *A*_*x*_ and *A*_*y*_ components of the vector potential of the pump pulse, respectively. Time-dependent *x* (magenta line) and *y* (blue line) components of SAM dynamics under σ^+^ (c) and σ^–^ (d) pulses. For SAM, *x* (pink line) and *y* (olive line) components under the σ^+^ (e) and σ^–^ (f) pulse excitations are shown.

We will begin by addressing the *z* component, denoted as *S*_*z*_ and *L*_*z*_, of the SAM and the OAM, which are conventionally regarded as the primary contribution to demagnetization and angular momentum transfer. In Figure 1a,b, it is evident that both σ^+^ and σ^–^ light induce a reduction in the *z* component of both SAM and OAM. Notably, SAM and OAM exhibit distinctive responses to optical helicity (σ^+^ and σ^–^). As depicted in Figure 1a, we clearly discern optical helicity-dependent orbital dynamics. For σ^+^ pulse, we can see *L*_*z*_ decrease as the laser pulse reaches its peak at around 18 fs, followed by a very slow increase until the pulse end. In contrast, for the σ^–^ pulse, the *L*_*z*_ continues to decrease until the end of the pulse. Interestingly, normalized OAM, defined as *L*_*z*_(*t*)/*L*_*z*_(0) – 1, is obviously faster than SAM, *S*_*z*_(*t*)/*S*_*z*_(0) – 1, for both σ^+^ and σ^–^ pulses, as illustrated in Figure 1a,d. This observation agrees well with experimental results and theoretical calculations by employing a linearly polarized laser.^21,22^

We now turn our attention to the *x* and *y* components (defined as *L*_*x*_, *L*_*y*_, *S*_*x*_, and *S*_*y*_) of both SAM and OAM. In their ground states, *L*_*x*_, *L*_*y*_, *S*_*x*_, and *S*_*y*_ are minimal values. OAM was generally quenched due to the motion of electrons in the lattice. Interestingly, our results unequivocally demonstrate that a circularly polarized laser pulse induces substantial enhancements in the *x* and *y* components of both SAM and OAM for Fe, Co, and Ni, as depicted in Figure 1c–f (refer to Figures S1 and S2 for Fe and Ni). These metals manifest analogous responses when subjected to σ^+^ and σ^–^ pulses. From Figure 1, we generally observed that *L*_*x*_, *L*_*y*_, *S*_*x*_, and *S*_*y*_ demonstrate significant rapid oscillations associated with the frequency of the laser (*f*_laser_), and *L*_*x*_, *L*_*y*_, *S*_*x*_, and *S*_*y*_ show a general increase in the amplitude of oscillation after laser pulse excitation peaking at the culmination of the pump pulse. Subsequently, the oscillations begin to decay, ultimately leading to disorganized behavior following the pulse concludes. For OAM, the decay for amplitudes of *L*_*x*_ and *L*_*y*_ oscillation induced by both σ^+^ and σ^–^ pulses are nearly identical. However, for SAM, the *S*_*x*_ and *S*_*y*_ manifest strong helicity-dependent oscillation behavior. Specifically, σ^+^ induced oscillated amplitude of *S*_*x*_ and *S*_*y*_ quickly decay, while those induced by σ^–^ exhibit a notably slower decay. Importantly, we note that the OAM amplitudes are approximately 20 times larger than those of SAM, demonstrating that the orbital degree of freedom of electrons has a stronger optical response than that of spin. Figure 1 clearly illustrates that the oscillations of the *x* and *y* components precede the reduction of the *z* component of both SAM and the OAM. These results indicate that the *x* and *y* components play an important role in the early stages of demagnetization dynamics.

The *x* and *y* components of both spin and orbital angular momenta for Fe, Co, and Ni exhibit a regular oscillation, strongly suggesting an optical helicity-driven precession. Figure 2 provides visual evidence of this phenomenon (refer to Figures S3 and S4 for Fe and Ni) where σ^–^ light induces a regular left-handed (LH) helix, while σ^–^ light induces a corresponding right-handed (RH) helix in the *x* and *y* components of spin and orbital angular momentum. Notably, these helices originate from the origin and steadily increase in amplitude until the laser pulse reaches its peak intensity. Subsequently, the helices tend to return to the origin: we also can observe the oscillatory decay in the *x* and *y* components of spin and orbital angular momenta as shown in Figure 1. Furthermore, our observations indicate that the σ^+^ pulse induces a larger amplitude of precession in spin compared to the precession induced by the σ^–^ pulse. That means the spin dynamics show the optical helicity-dependent precession. We can also see that the amplitude of orbital for precession is significantly larger than that of spin, which suggest that OAM will has the larger obliquity of precession than SAM. These findings reveal physical pictures of early dynamics of SAM and OAM under the influence of circularly polarized light, which is important for understanding ultrafast demagnetization processes in magnetic materials.

**Figure 2:**
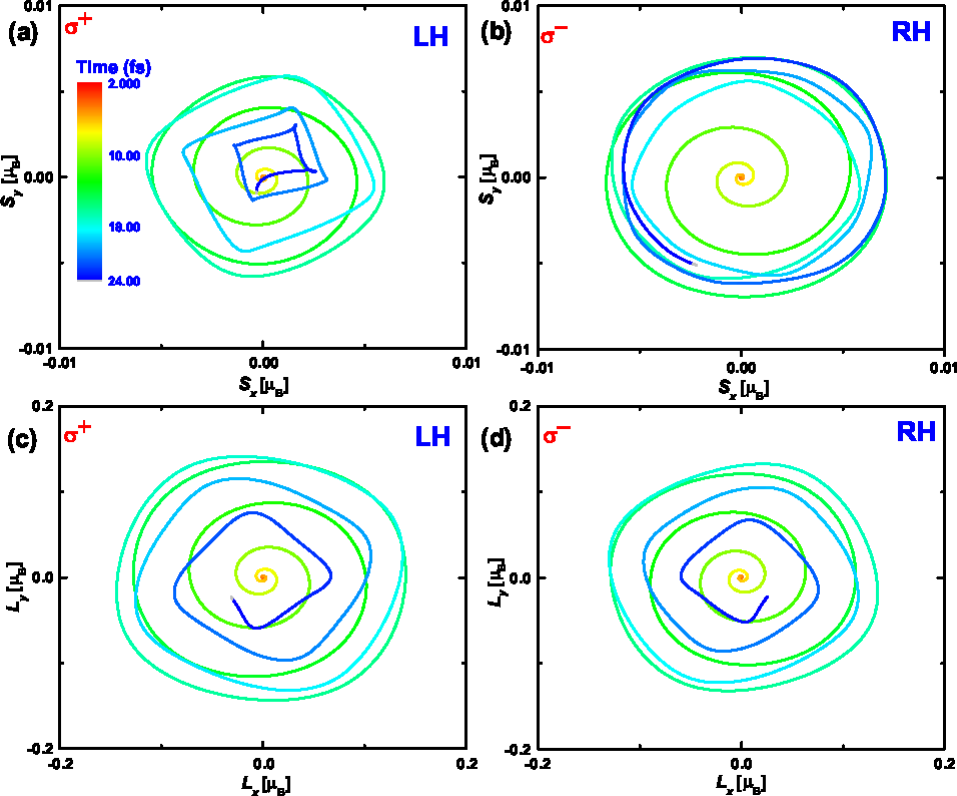
Left-handed (LH) and right-handed (RH) precessions of SAM and OAM induced by circularly polarized pulse. Panels (a) and (b) depict the LH and RH precession of the SAM under σ^+^ and σ^–^ pulses, respectively. Panels (c) and (d) illustrate the LH and RH precession of the OAM under σ^+^ and σ^–^ pulses, respectively. The color maps indicate the time scale from the start to the end of the pulse.

The precession of both **L** and **S** results from a torque (**T**) induced by the laser, which can be written as  and  for spin and orbital, respectively. The direction-dependent torque is plotted as a function of time in Figure 3a. The pulse induces a significant torque for the *x* and *y* components of SAM, while the *z* component of the OAM and all directions of the SAM exhibit minimal torque. This observation aligns with the aforementioned results for the pronounced magnitudes of *L*_*x*_ and *L*_*y*_ in Figure 1. The *x* and *y* components of torque for both **L** and **S** regularly oscillate, corresponding to the precession behavior of **L** and **S**. Subsequently, we further calculated time-dependent obliquity dynamics, as illustrated in Figure 3. For **L**, during the early time from 0 to 8 fs, the obliquity (θ_*L*_) of the OAM precession exhibits a slow increase and then from 8 to 18 fs exponentially enhances to the maximum saturate value (around 70°). The helicity-dependent dynamics of θ_*L*_ can also be clearly seen in Figure 3b: the enhancement induced by σ^+^ surpasses that induced by σ^–^. However, σ^+^ and σ^–^ induce only a minute spin obliquity (θ_*S*_). Figure 3c demonstrates that the maximum saturation value of θ_*S*_ is around 0.5°. Despite σ^+^ inducing a larger θ_*S*_ angle than σ^–^, the θ_*S*_ angle is 2 orders of magnitude smaller than θ_*L*_. Consequently, θ_*S*_ is unlikely to substantially influence the obliquity (θ_*J*_) of the total angular momentum, as evident in Figure 3d. Notably, the trends for θ_*J*_ and θ_*L*_ exhibit remarkably similar behavior, with both σ^+^-induced θ_*J*_ and θ_*L*_ being larger than those of σ^–^. On the other hand, even though θ_*L*_ is considerable, the magnitude of **L** is markedly smaller than that of **S**, leading to a relatively moderate θ_*J*_ value according to vector sum. Overall, the difference between θ_*L*_ and θ_*J*_ from two helicities is much more pronounced for σ^+^ pulses compared to that of σ^–^ pulses. We also compared the demagnetization and θ_*J*_ dynamics, as depicted in Figure 3d. We found that the initiation time for angular momentum precession (beginning at around 5 fs) is evidently earlier than that of demagnetization (coming around 10 fs).

**Figure 3:**
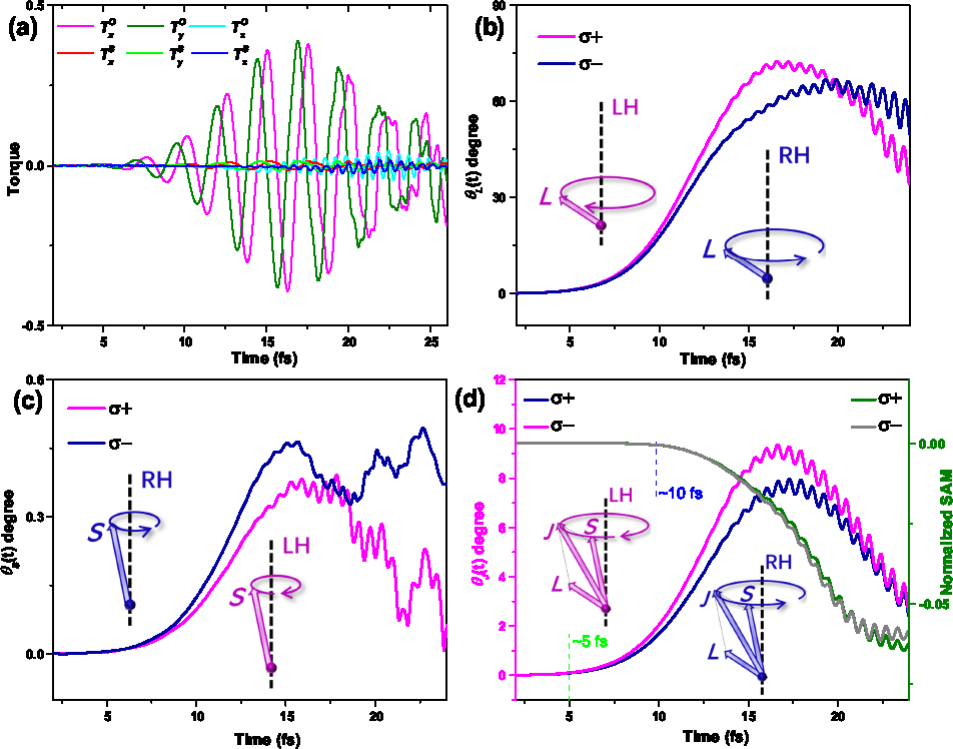
Laser-induced torques and obliquity of precession for **L**, **S**, and **J**. (a) Direction-resolved torque for *x*, *y*, and *z* components of SAM and OAM. Time-dependent dynamics of precession obliquity for (b) SAM θ_*S*_, (c) OAM θ_*L*_, and (d) total angular momentum θ_*J*_ induced by σ^+^ (purple line) and σ^–^ (blue line) laser pulse. Inserted schematic illustrations represent the σ^+^ and σ^–^ laser pulse induced left-handed (LH) and right-handed (RH) precession of **L**, **S**, and **J**, respectively. The green and blue dashed lines with marks at 5 and 10 fs denote the estimated initial times for precession and demagnetization, respectively.

Next, we will examine how changing the laser parameters affects the dynamics of SAM and OAM. First, If we look at a longer pulse with FWHM =26.6 fs, the SAM and the OAM will have precession in a longer time, as shown in Figure 4d–g. Moreover, from Figure 1, we can clearly see that the frequencies (ω_*p*_) of SAM and the OAM precession essentially coincide with the applied laser frequency (*f*_laser_) of 395 THz (1.63 eV). Therefore, we will investigate whether the ω_*p*_ of SAM and OAM depend on the *f*_laser_. We consider different incident *f*_laser_, including 66, 197, 395, 658, and 1316 THz, where the fluence and duration of the laser were fixed as shown in Figure 4. Here, the determination of ω_*p*_ for the SAM and the OAM is accomplished through Fourier transformation of their respective temporal dynamics. For Figure 4a, as *f*_laser_ increases, we can observe that both ω_*p*_ values of **L** and **S** increase linearly, and the ω_*p*_ values for **S** and **L** are almost identical. Employing a linear regression analysis, it is established that the precession frequencies for **S** and **L** are in direct proportion to the incident *f*_laser_.

**Figure 4:**
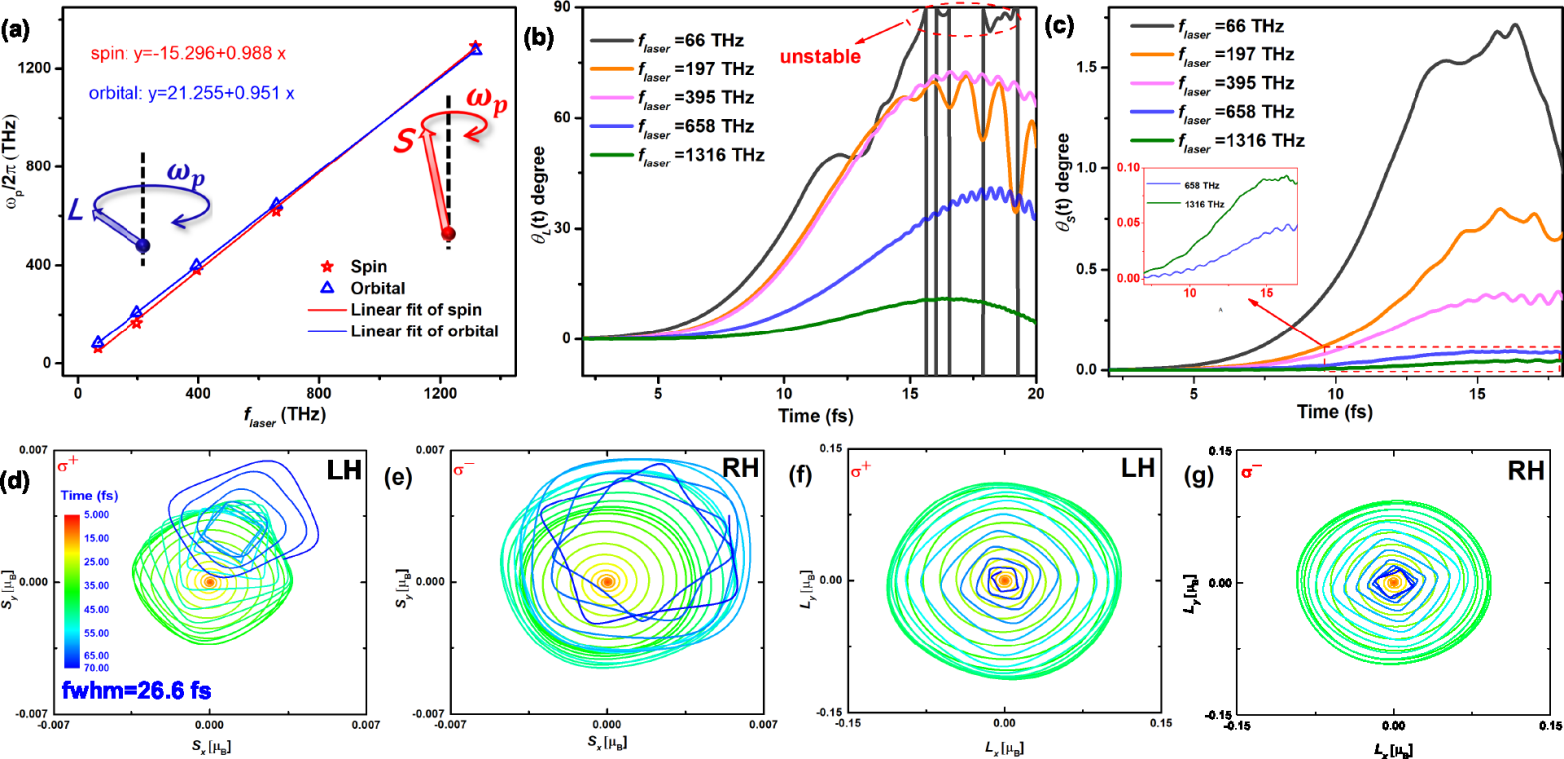
Dynamics of SAM and OAM affects by changing laser parameters. (a) The relationship between laser frequency (*f*_laser_) and the frequency (ω_*p*_/2π) of precession for SAM and OAM. Inserted schematic illustrations represent the precession angular frequency (ω_*p*_) of **L** and **S**, respectively. Frequency-dependent precession obliquities for (b) θ_*S*_ and (c) θ_*L*_ are displayed. The unstable state in low frequency *f*_laser_ = 66 THz is also indicated in (b). Panels (d) and (e) depict the LH and RH precession of SAM under σ^+^ and σ^–^ pulses with FWHM = 26.6 fs, respectively. Panels (f) and (g) illustrate the LH and RH precessions of the OAM under σ^+^ and σ^–^ pulses with the same parameter, respectively. The color maps indicate the time scale from the start to the end of the pulse.

To further analyze the frequency dependence of SAM and OAM dynamics, we calculated the obliquity (θ_*S*_ and θ_*L*_) of SAM and OAM within different *f*_laser_ ranging from 66 to 1316 THz (see Figure 4b,c). Our findings show that the higher *f*_laser_ laser pulse generally leads to a notable reduction in θ_*S*_ and θ_*L*_, while conversely, the lower *f*_laser_ laser pulses tend to yield larger values of θ_*S*_ and θ_*L*_. Notably, for the OAM, as *f*_laser_ reaches 1316 THz, θ_*L*_ gradually increases from 0° to about 10°, and at this frequency, it can give rise to a θ_*L*_ approaching 90°. Because the *f*_laser_ is directly proportional to the ω_*p*_ of SAM and OAM, lower frequency lasers result in a slower ω_*p*_, thereby leading to an increase in the precession angle. Such behavior parallels that of classical gyroscope precession, where a decrease in precession angular frequency leads to an increase in precession angle. Consequently, when a sufficiently high *f*_laser_ laser pulse is applied, it drives the precession angle to toward 90°, resulting in an unstable precession. In the case of gyroscope precession, an increase in the precession angle or a decrease in ω_*p*_ also leads to an unstable motion in precession. With regard to SAM, we similarly observe a substantial influence of *f*_laser_ on the θ_*S*_. However, unlike the OAM, the SAM only exhibits a small precession angle. For example, a high *f*_laser_ of 1316 THz yields a maximum θ_*S*_ of merely 0.05°. While a low *f*_laser_ can enhance the θ_*S*_ by approximately 30-fold, reaching 1.5°, it remains smaller in comparison to that of the OAM. Our finding illustrates that the *f*_laser_ can effectively manipulate the precession of both the SAM and the OAM.

Previous studies have primarily focused on analyzing the dynamics of the *z* component of angular momentum, regarding them as the primary factors contributing to both demagnetization and angular momentum transfer. However, the *x* and *y* components of both the SAM and the OAM, which have received comparatively less attention, may exert significant influence over magnetization dynamics. Recent theoretical works have showcased distinct spin dynamics in magnetic or nonmagnetic materials induced by a circularly polarized laser pulse;^31−33^ however, chiral spin precession and oscillated transverse magnetism have not been reported to date. Notably, our findings demonstrate that a circularly polarized laser pulse induces a considerably larger component of the OAM compared to the SAM, signifying a more robust optical response from the OAM. As illustrated in Figure S5, the OAM exhibits a stronger optical response than the SAM with changes in the pulse amplitude. These results suggest that the angular momentum of a circularly polarized laser may predominantly transfer to the orbital component, driving spin precession through the SOC. The dynamics of *x* and *y* components of the SAM and the OAM in Co are shown for SOC scaled by factors of 1.5 and 2.0 (see Figure S6). It is evident that an increase in SOC leads to an increased SAM but no change in OAM. Moreover, Furthermore, it is noteworthy that theoretical calculations have reported the induction of large OAM components of electrons through femtosecond laser pulses in Co/Cu(100) interfaces,^34,35^ Pt films,^36^ and metallic clusters.^37^ Consequently, the significant generation of OAM components by light demonstrates considerable potential in the emerging field of *orbitronics*.^23,25^

The OISTR effect proposes that light can directly and coherently interact with spin, representing the fastest means of controlling spin. This is achieved through light-induced spin-selective charge excitation within the sublattice of magnetic materials. Our results demonstrate that the time scale of OAM and SAM precession occurs extremely rapidly (<10 fs), even preceding that of the OISTR effect and demagnetization. This is supported by the corresponding time scales of the *x* and *y* components of SAM and OAM, as illustrated in Figures 1c–f and 3. The orientation of SAM and OAM plays a crucial role in comprehending the microscopic mechanisms involved in laser-induced demagnetization.

Moreover, it is important to note that our simulations were specifically focused on the early spin dynamics, thus limiting the time scale to within 100 fs. However, in the time scale of 50–100 fs, electron–phonon coupling will play a crucial role in influencing the precession of SAM and OAM, leading to a complex angular momentum transfer involving phonons. Recently, Tauchert et al.^38^ observed *circularly polarized phonons* or *chiral phonons* in the demagnetization process due to angular momentum transfer from spin systems. The angular momentum transfer between polarized phonon and chirality of SAM and OAM dynamics presents an intriguing open question warranting further investigation.

In summary, we employed rt-TDDFT simulations to explore the SAM and OAM dynamics in ferromagnetic Fe, Co, and Ni, subjected to circularly σ^+^ and σ^–^ polarized laser pulses. We unveiled pronounced *x* and *y* components for both SAM and OAM, with the OAM components exhibiting magnitudes that are an order of magnitude larger than their SAM counterparts. This observation emphasizes a more substantial optical response emanating from the electrons’ orbital degrees of freedom in comparison to their spin. Furthermore, we noted a clear dependence of the *x* and *y* of component oscillations on the optical helicity. Intriguingly, σ^+^ and σ^–^ pulses were observed to induce distinct chiralities in the precession of SAM and OAM: the σ^+^ pulse results in regular LH helical dynamics, while the σ^–^ pulse fosters a RH helical dynamics of both SAM and OAM. These chiral precession dynamics show a strong correlation with the laser’s frequency and duration. Our results reveal an exceptionally rapid precession of the OAM and SAM, outstripping the time scale of the demagnetization process and the OISTR effect. Such chirality of spin and orbital dynamics could be important for circularly polarized phonons in the demagnetization process.

## Supporting Information Available

Computational methods, *x* and *y* components of SAM and OAM dynamics for Fe and Ni atoms, left-handed (LH) and right-handed (RH) precession of SAM and OAM for Fe and Ni, dependence of the dynamics of *x* and *y* components of SAM and OAM with changing amplitude of pulse and SOC (PDF)
