# Supplementary material for: Ultrafast Chiral Precession of Spin and Orbital Angular Momentum Induced by Circularly Polarized Laser Pulse in Elementary Ferromagnets
Source: J Phys Chem Lett. 2024 Feb 26;15(9):2493–8. doi: 10.1021/acs.jpclett.4c00291 (PMC10926150; doi:10.1021/acs.jpclett.4c00291)
Supplement: Supplementary file 1 — jz4c00291_si_001.pdf [file jz4c00291_si_001.pdf]

Supplementary Materials "**Ultrafast chiral precession of spin and orbital angular momentum induced by circularly polarized laser pulse in elementary ferromagnets**"

Junjie He[1], Thomas Frauenheim[2], Shuo Li[3],

[1] *Department of Physical and Macromolecular Chemistry, Faculty of Science, Charles University, Prague 12843, Czech Republic*

[2]*Bremen Center for Computational Materials Science, University of Bremen, Bremen 28359, Germany*

[3]*Institute of Advanced Study, Chengdu University, Chengdu 610100, China*

E-mail: junjie.he@natur.cuni.cz; shuoli.phd@gmail.com

Computational methods

We employ the real-time time-dependent density functional theory (rt-TDDFT) to study the spin and orbital dynamics of Fe, Co and Ni metals. The foundation of rt-TDDFT is the Runge–Gross theorem, [1] which posits that a time-dependent external potential uniquely defines the functional of time-dependent density, contingent upon the initial state. Based on this theorem, a system of non-interacting electron can be chosen such that the density of this non-interacting system is equal to that of the interacting system for all times with the wave function of this non-interacting system represented by a Slater determinant of single-particle orbitals. In a fully non-collinear spin-dependent version of these theorems, time-dependent Kohn–Sham (KS) orbitals are Pauli spinors governed by the Schrodinger equation:

$$i\frac{\partial\psi_j(\mathbf{r},t)}{\partial t} = \left[\frac{1}{2}\left(-i\nabla + \frac{1}{c}\mathbf{A}_{\mathbf{ext}}(t)\right)^2 + \upsilon_s(\mathbf{r},t) + \frac{1}{2c}\sigma\cdot\mathbf{B}_{\mathbf{s}}(\mathbf{r},t) + \frac{1}{4c^2}\sigma\cdot(\nabla\upsilon_s(\mathbf{r},t)\times -i\nabla)\right]\psi_j(\mathbf{r},t) \quad (1)$$

where $\mathbf{A}_{\mathbf{ext}}(t)$ and σ represent a vector potential and Pauli matrices. The KS effective potential $\upsilon_s(\mathbf{r},t) = \upsilon_{ext}(\mathbf{r},t) + \upsilon_H(\mathbf{r},t) + \upsilon_{xc}(\mathbf{r},t)$ can be decomposed into the external potential υ_{ext}, the classical Hartree potential υ_H, and the exchange-correlation (XC) potential υ_{xc}, respectively. The KS magnetic field can be written as $\mathbf{B}_{\mathbf{s}}(\mathbf{r},t) = \mathbf{B}_{\mathbf{ext}}(\mathbf{r},t) + \mathbf{B}_{\mathbf{xc}}(\mathbf{r},t)$, where $\mathbf{B}_{\mathbf{ext}}$ and $\mathbf{B}_{\mathbf{xc}}$ represent the magnetic field of the applied laser pulse plus possibly an additional magnetic field and XC magnetic field, respectively. The last term in Eq. (1) stand for the SOC. We only time propagate the electronic system while keeping the nuclei fixed.

All calculations were employed by a fully non-collinear spin version[2] of rt-TDDFT by implementing through the full-potential augmented plane-wave ELK code.[3] We utilized a 12 × 12 × 1 regular mesh in k-space, and the rt-TDDFT simulations were conducted with a time step of Δt = 0.1 atomic units. The smearing width was set to 0.027 eV. The laser pulses employed in our analyses were circularly polarized. Additionally, all calculations adhered to the adiabatic local spin density approximation (ALSDA), consistent with methodologies established in previous works

The circularly polarized fields can be written as $\boldsymbol{A} = (A_x, A_y, 0)$,

$$A_x(t) = \begin{cases} A_0 \cos(\omega t) \sin\left(\frac{\pi t}{T}\right), & 0 \leq t \leq T \\ 0, & otherwise \end{cases} \tag{2}$$

$$A_y(t) = \begin{cases} A_0 \cos(\omega t - \varphi) \sin\left(\frac{\pi t}{T}\right), & 0 \leq t \leq T \\ 0, & otherwise \end{cases} \tag{3}$$

where φ is the polarization angle and φ = ±90° are used for circularly σ^+ and σ^- polarized laser pulses. The second sinusoidal term of Eq. (2) and Eq. (3) constitutes the temporal envelope of the pulse, in which T is a duration.

In general, the OAM operator $L = r \times p$ cannot be strictly defined because the position operator r cannot be well defined in a periodic solid[4]. However, if the angular momentum arises from orbitals localized within the muffin tin (i.e., a sphere around the point nuclei), which should be as large as possible. This approach can still be employed: one sets r = 0 as the center of the muffin tin and performs the integral of the L expectation value only within this sphere. The basic assumption for the transient case is that the current loops induced by laser pulses are predominantly contained within the muffin tin[5]. The low fluence of laser pulses is performed for the validity of OAM. Our calculation of the expectation of OAM for ferromagnetic metals was based on the previous work in Ref.6

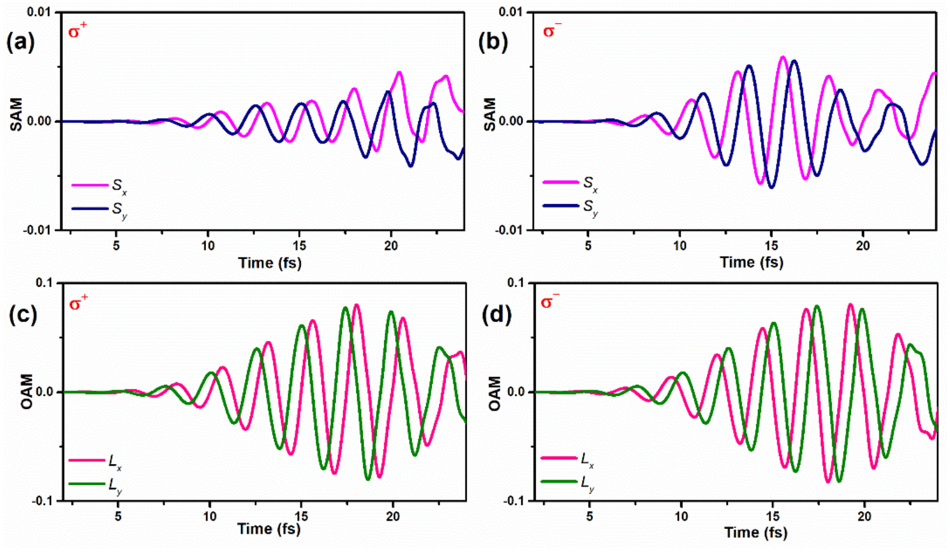

Figure S1: The x and y components of SAM and OAM dynamics for Fe under excitation by σ^+ and σ^- pulse, with the pulses being circularly polarized and characterized by fwhm of 9.68 fs, a central frequency of 1.63 eV, and an incident fluence of 7.1 mJ/cm². Time-dependent x (magenta line) and y (blue line) components of SAM dynamics under the σ^+ (a) and σ^- (b) pulses excitations. The x (pink line) and y (olive line) components of OAM under σ^+ (c) and σ^- (d) pulse excitations.

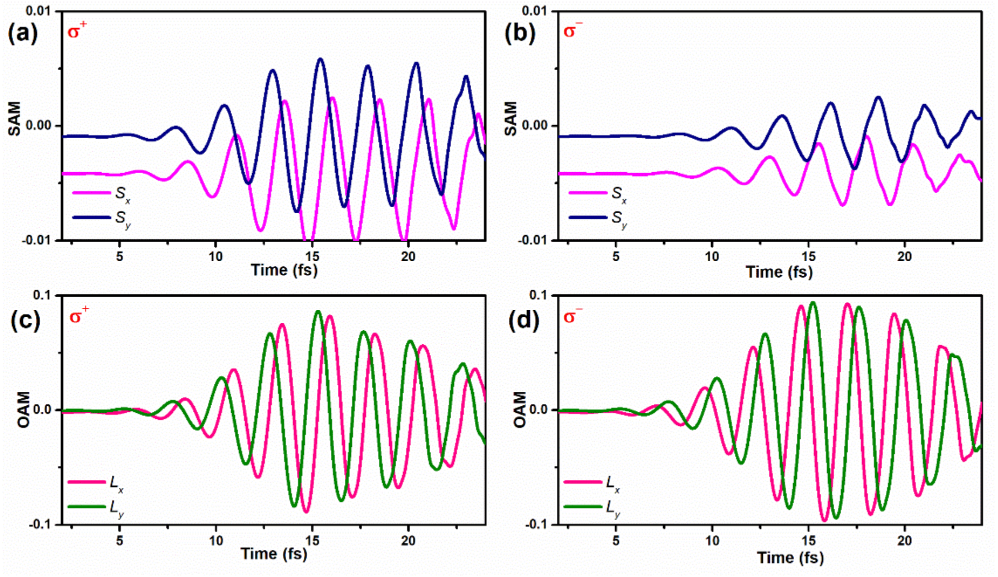

Figure S2: The x and y components of SAM and OAM dynamics for Ni under excitation by σ^+ and σ^- pulse, with the pulses being circularly polarized and characterized by fwhm of 9.68 fs, a central frequency of 1.63 eV, and an incident fluence of 7.1 mJ/cm². Time-dependent x (magenta line) and y (blue line) components of SAM dynamics under the σ^+ (a)

and σ^- (b) pulses excitations. The x (pink line) and y (olive line) components of OAM under σ^+ (c) and σ^- (d) pulse excitations.

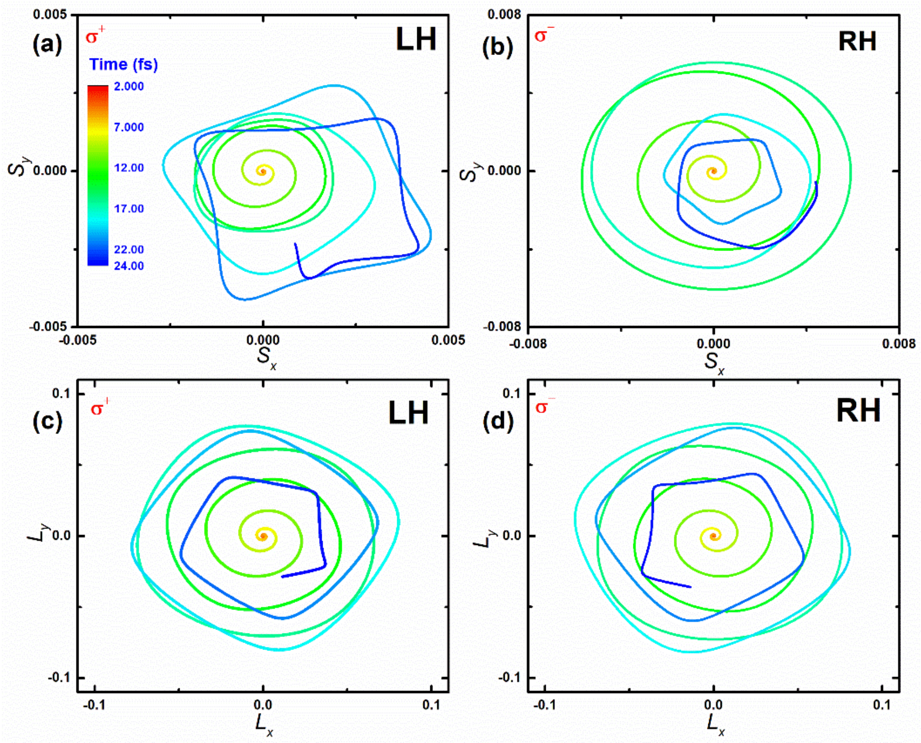

Figure S3: Left-handed (LH) and right-handed (RH) precession of SAM and OAM for Fe induced by circularly polarized pulse. Panels (a) and (b) depict the LH and RH precession of SAM under σ^+ and σ^- pulses, respectively. Panels (c) and (d) illustrate the LH and RH precession of OAM under σ^+ and σ^- pulses, respectively. The color maps indicate the time scale from the start to the end of the pulse.

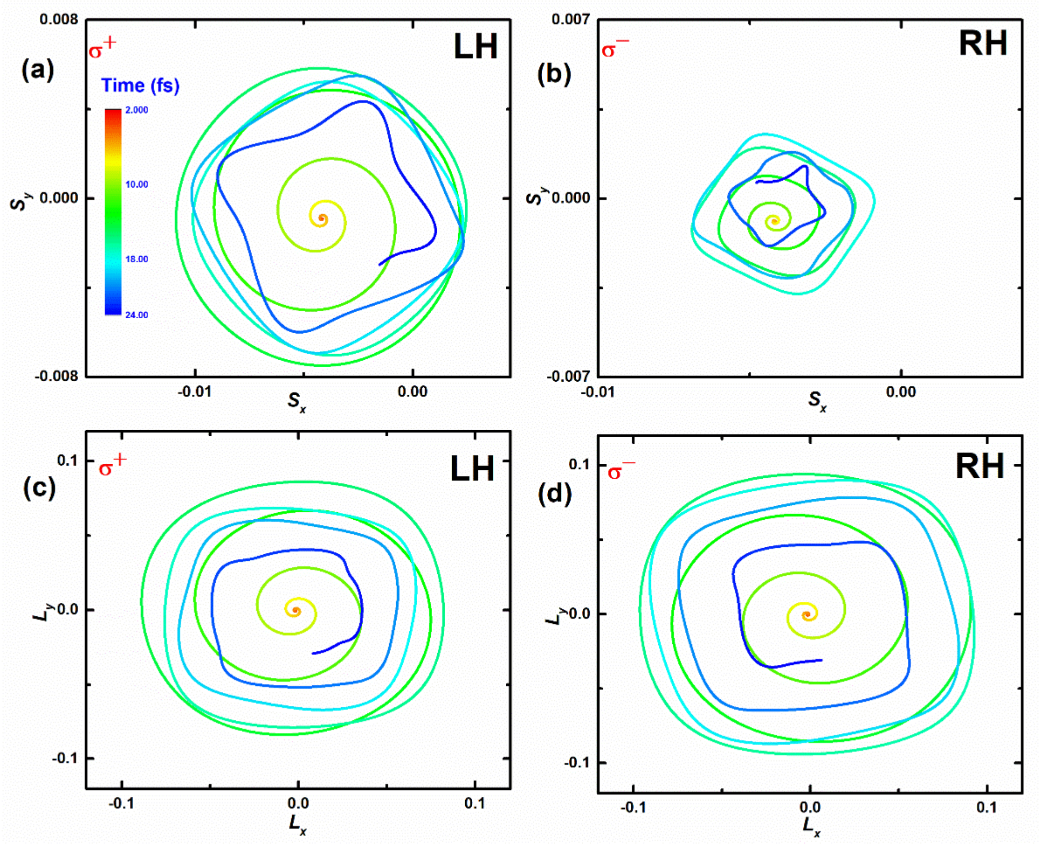

Figure S4: Left-handed (LH) and right-handed (RH) precession of SAM and OAM for Ni induced by circularly polarized pulse. Panels (a) and (b) depict the LH and RH precession of SAM under σ^+ and σ^- pulses, respectively. Panels (c) and (d) illustrate the LH and RH precession of OAM under σ^+ and σ^- pulses, respectively. The color maps indicate the time scale from the start to the end of the pulse.

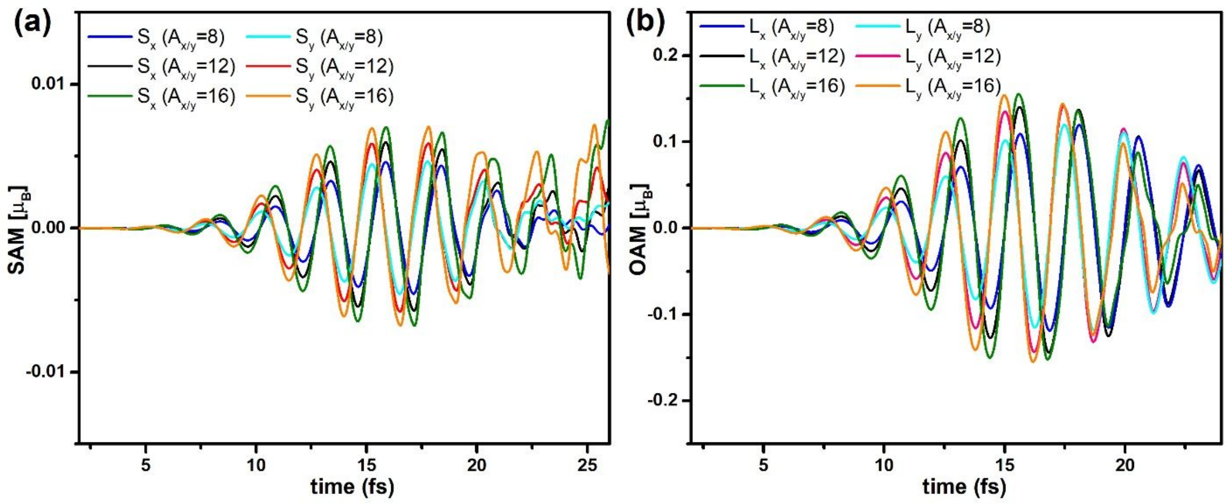

Figure S5. Dependence of the dynamics of *x* and *y* component of SAM (a) and OAM (b) amplitude of pulse. The SAM and OAM dynamics are shown for amplitude with 8, 12 and 16 respectively.

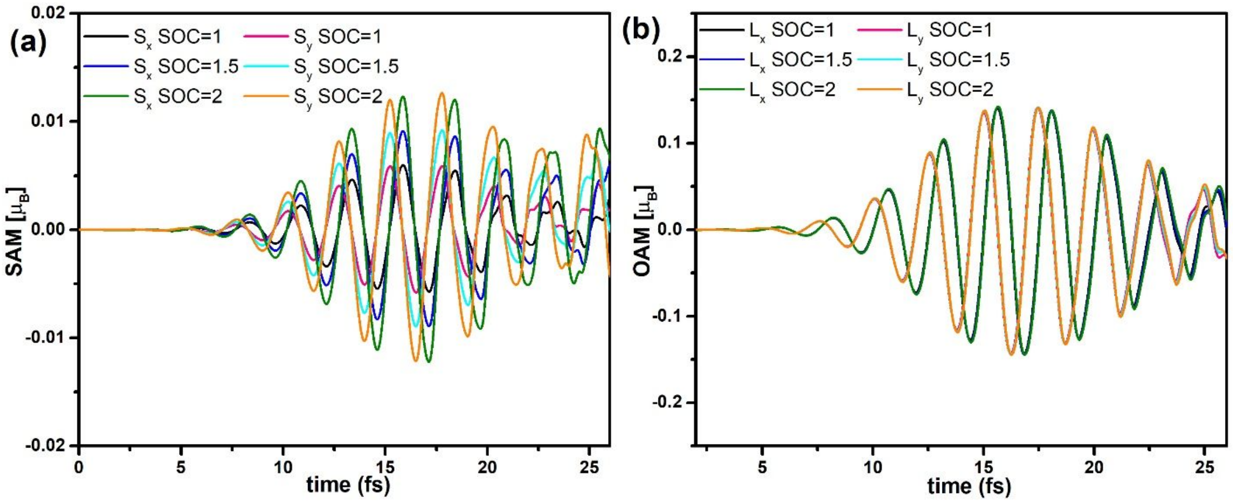

Figure S5. Dependence of the dynamics of *x* and *y* component of SAM (a) and OAM (b) on the spin-orbit coupling (SOC) constant. The SAM and OAM dynamics are shown for SOC scaled by factors of 1, 1.5 and 2.0.

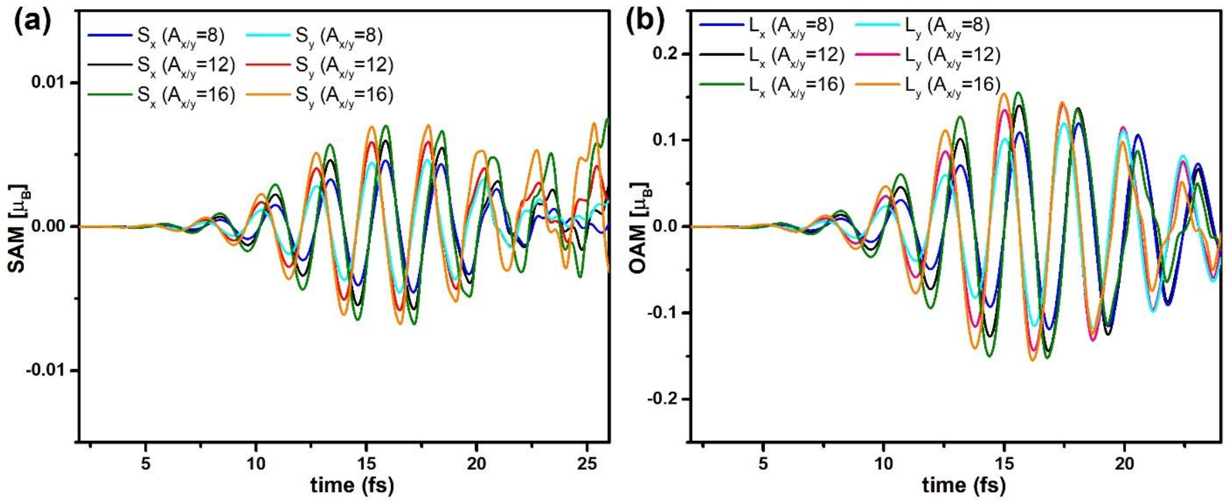

Figure S6. Dependence of the dynamics of *x* and *y* component of SAM (a) and OAM (b) amplitude of pulse. The SAM and OAM dynamics are shown for amplitude with 8, 12 and 16 respectively.

Reference:

---

[1] Runge E.; Gross, E. K. U. *Phys. Rev. Lett.* **1984**, *52*, 997.

[2] Krieger, K.; Dewhurst, J. K.; Elliott, P.; Sharma, S.; Gross, E. K. U. *J. Chem. Theory Comput.* **2015**, 11, 4870.

[3] The ELK code, http://elk.sourceforge.net.

[4] Resta, R. Magnetic circular dichroism versus orbital magnetization. *Phys. Rev. Res.* **2020**, *2*, 023139.

[5] Elliott, P.; et al. The microscopic origin of spin-orbit mediated spin-flips. *J. Magn. Magn. Mater.* **2020**, *502*, 166473.

[6] Dewhurst, J.; et al. Angular momentum redistribution in laser-induced demagnetization. *Phys. Rev. B* **2021**, *104*, 054438.
